# IMGD: an integrated platform supporting comparative genomics and phylogenetics of insect mitochondrial genomes

**DOI:** 10.1186/1471-2164-10-148

**Published:** 2009-04-07

**Authors:** Wonhoon Lee, Jongsun Park, Jaeyoung Choi, Kyongyong Jung, Bongsoo Park, Donghan Kim, Jaeyoung Lee, Kyohun Ahn, Wonho Song, Seogchan Kang, Yong-Hwan Lee, Seunghwan Lee

**Affiliations:** 1 Insect Biosystematics Laboratory, Seoul National University, Seoul 151–921, Korea; 2 Research Institute for Agricultural and Life Sciences, Seoul National University, Seoul 151–921, Korea; 3 Department of Agricultural Biotechnology, Seoul National University, Seoul 151–921, Korea; 4 Fungal Bioinformatics Laboratory, Seoul National University, Seoul 151–921, Korea; 5 Center for Fungal Pathogenesis, Seoul National University, Seoul 151–921, Korea; 6 Center for Fungal Genetic Resources, Seoul National University, Seoul 151–921, Korea; 7 Department of Plant Pathology, Penn State University, University Park, PA 16802, USA; 8 Center for Agricultural Biomaterials, Seoul National University, Seoul 151–921, Korea

## Abstract

**Background:**

Sequences and organization of the mitochondrial genome have been used as markers to investigate evolutionary history and relationships in many taxonomic groups. The rapidly increasing mitochondrial genome sequences from diverse insects provide ample opportunities to explore various global evolutionary questions in the superclass Hexapoda. To adequately support such questions, it is imperative to establish an informatics platform that facilitates the retrieval and utilization of available mitochondrial genome sequence data.

**Results:**

The Insect Mitochondrial Genome Database (IMGD) is a new integrated platform that archives the mitochondrial genome sequences from 25,747 hexapod species, including 112 completely sequenced and 20 nearly completed genomes and 113,985 partially sequenced mitochondrial genomes. The Species-driven User Interface (SUI) of IMGD supports data retrieval and diverse analyses at multi-taxon levels. The Phyloviewer implemented in IMGD provides three methods for drawing phylogenetic trees and displays the resulting trees on the web. The SNP database incorporated to IMGD presents the distribution of SNPs and INDELs in the mitochondrial genomes of multiple isolates within eight species. A newly developed comparative SNU Genome Browser supports the graphical presentation and interactive interface for the identified SNPs/INDELs.

**Conclusion:**

The IMGD provides a solid foundation for the comparative mitochondrial genomics and phylogenetics of insects. All data and functions described here are available at the web site .

## Background

The mitochondrial genomes of members of the superclass Hexapoda (generally referred to as the 'insects') are typically approximately 15 kilobases (kb) in length and encode 37 genes, including 13 protein coding genes (PCGs), 2 ribosomal RNA genes (rRNAs), and 22 transfer RNA genes (tRNAs). Owing to its small size, high copy number, and relatively infrequent gene rearrangements, the mitochondrial genome has been extensively used for phylogenetic analyses [1-4]. Phylogenetic analysis based on the mitochondrial gene sequences is often limited to closely related species, due to the high rate of nucleotide substitutions. However, variations in the mitochondrial gene content and order have been utilized to elucidate evolutionary relationships among distantly-related species, on the basis of shared derived characteristics that denote the common ancestry of a given group [5].

Recent years, the number of sequenced mitochondrial genomes has been increasing fast due to rapidly growing sequencing capacity [6]. For example, more than 1,200 metazoan mitochondrial genomes have already been sequenced completely [7,8]. The abundance of available mitochondrial genomes has led to the development of the following web-based relational databases that are specialized for archiving the resulting data: GObase [9], AMiGA [10], Mitome [8], MamMibase [11], OGRe [7], and NCBI Organelle Genome Resources [12]. Some of these resources also provide tools for data analysis and/or viewing: MamMibase provides a web-based phylogenetic analysis tool for studying evolutionary relationships on the basis of the archived mitochondrial genomes [11] and Mitome provides a graphical mitochondrial genome browser [8]. In order to more effectively support uses of the rapidly accumulating mitochondrial genome sequences, an integrated platform that provides a diverse array of analysis tools is necessary.

Single nucleotide polymorphisms (SNPs) in the insect mitochondrial genome sequences can support fine-scale phylogenetic analyses, as illustrated in the following examples. Twenty-four biotypes of *Bemisia tabaci *(Hemiptera: Aleyrodidae), which could not be distinguished by morphological characteristics, were resolved [13]. SNPs in the cytochrome c oxidase subunits I (*COI*) locus of *Aedes aegypti *(Diptera: Cuclicidae) were used to differentiate four strains [14]. Based on the fully sequenced mitochondrial genomes in the genus *Flustrellidra *(Ctenostomata: Flustrellidridae), a SNP analysis was conducted to identify a suitable gene maker for distinguishing morphologically similar species [15].

Partially sequenced mitochondrial genes from a very large number of species also provide valuable markers for phylogenetic analysis. For example, the *COI *gene has been used extensively for species identification in the 'DNA barcoding' projects [16,17]. In particular, in Hexapoda, DNA barcoding projects covering multiple orders have been conducted [18-21], resulting in at least 50,000 partial sequences of *COI *loci in the NCBI. Because the cytochrome c oxidase subunits II (*COII*) locus is relatively small (approximately 600 bp) and can be amplified well by PCR from diverse species [22,23], many researchers have sequenced this locus, yielding more than 15,000 sequences from Hexapoda. Due to the large number of characterized insect species, sequences of these loci are an excellent resource for comprehensive phylogenetic analyses of insects; however, such data have not yet been archived in the currently available mitochondrial genome databases.

A new integrated platform named the Insect Mitochondrial Genome Database (IMGD; ) was developed to better integrate available mitochondrial gene and genome sequences and to provide bioinformatics tools for efficient data retrieval and utilization. The IMGD archives the sequences of 112 completely sequenced and 20 nearly completed mitochondrial genome sequences, as well as partial sequences of 113,985 mitochondrial genomes (Tables 1, 2, 3) from 25,747 insect species using the standardized framework of the Comparative Fungal Genomics Platform (CFGP; ) [24]. SNPs in the mitochondrial genomes of multiple isolates within eight species were identified via the SNP Analysis Platform (SAP; ; J. Park *et al*., unpublished) and presented through the SNU Genome Browser () [25]. BLAST [26], tRNAScan-SE [27], and mFold [28] were also incorporated into IMGD. Additionally, three phylogenetic analysis tools, including ClustalW, PHYML, and PHYLIP [29-31], were integrated into IMGD to facilitate analyses across multiple species: these tools are available through the web interface supported by Phyloviewer (; B. Park *et al*., unpublished). To assist the comparison of these sequences and phylogenetic analysis within selected taxa, a new user interface, termed the Species-driven User Interface (SUI), was designed and implemented. The IMGD provides a highly integrated environment for conducting evolutionary studies of insects using their mitochondrial gene/genome sequences.

**Table 1:** List of the number of mitochondrial sequences in Hexapoda archived in the IMGD

**Order**	**Species**	**CG^a^**	**NG^b^**	**PG^c^**
Archaeognatha	10	4	0	15
Blattaria	274	1	0	991
Coleoptera	6,594	8	4	25,783
Collembola	133	6	2	947
Dermaptera	35	0	0	63
Diplura	10	3	0	24
Diptera	3,846	24	2	26,982
Embioptera	14	0	0	26
Ephemeroptera	288	1	0	742
Grylloblattodea	17	0	1	113
Hemiptera	1,851	20	5	7,299
Hymenoptera	4,144	3	2	14,737
Isoptera	647	7	0	2,842
Lepidoptera	4,556	7	2	19,380
Mantodea	188	1	0	717
Mantophasmatodea	17	1	0	194
Mecoptera	61	0	0	141
Megaloptera	7	2	0	409
Neuroptera	143	2	0	437
Odonata	525	0	1	1,734
Orthoptera	919	14	0	4,744
Phasmatodea	64	0	1	482
Phthiraptera	527	3	0	2,155
Plecoptera	184	1	0	529
Protura	2	0	0	6
Psocoptera	121	1	0	338
Raphidioptera	4	0	0	5
Siphonaptera	35	0	0	134
Strepsiptera	6	0	0	7
Thysanoptera	154	1	0	857
Trichoptera	343	0	0	1,100
Zoraptera	1	0	0	2
Zygentoma	27	2	0	50

Total	25,747	112	20	113,985

^a^Completely sequenced mitochondrial genome, ^b^Nearly completely sequenced mitochondrial genome, and ^c^Partially sequenced mitochondrial gene sequences

**Table 2:** List of 56 whole mitochondrial genomes of hexapod species (Part I. 52 holometabolous species) archived in IMGD

**Order**	**Species**	**Size (bp)**	**GC (%)**	**PCGs**	**tRNAs**	**rRNAs**
Coleoptera	Chaetosoma scaritides*	15,511	20.96	13	22	2
Coleoptera	*Crioceris duodecimpunctata*	15,880	23.11	13	22	2
Coleoptera	*Cyphon *sp. BT0012	15,919	24.83	13	22	2
Coleoptera	Priasilpha obscura*	16,603	23.49	13	22	2
Coleoptera	*Pyrocoelia rufa*	17,739	22.59	13	22	2
Coleoptera	*Pyrophorus divergens*	16,120	30.56	13	22	2
Coleoptera	*Rhagophthalmus lufengensis*	15,982	20.37	13	22	2
Coleoptera	*Rhagophthalmus ohbai*	15,704	20.85	13	19	2
Coleoptera	*Sphaerius *sp. BT0074*	15,121	19.28	13	22	2
Coleoptera	*Tetraphalerus bruchi*	15,689	33.01	13	22	2
Coleoptera	Trachypachus holmbergi*	15,722	20.54	13	22	2
Coleoptera	*Tribolium castaneum*	15,881	28.32	13	22	2

Diptera	Anopheles funestus*	15,354	21.84	7	22	2
Diptera	*Anopheles gambiae*	15,363	22.44	13	22	2
Diptera	*Anopheles quadrimaculatus *A Orlando	15,455	22.64	13	22	2
Diptera	*Bactrocera oleae *Italy	15,815	27.41	13	22	2
Diptera	*Bactrocera oleae *Portugal	15,815	27.37	13	22	2
Diptera	*Ceratitis capitata*	15,980	22.52	13	22	2
Diptera	*Chrysomya putoria*	15,837	23.30	13	23	2
Diptera	*Cochliomyia hominivorax*	16,022	23.10	13	22	2
Diptera	*Cydistomyia duplonotata*	16,247	22.07	13	23	2
Diptera	*Drosophila ananassae*	14,920	22.59	13	22	2
Diptera	*Drosophila erecta*	14,952	22.77	13	22	2
Diptera	*Drosophila grimshawi*	14,874	23.24	13	22	2
Diptera	*Drosophila mauritiana *G52	14,964	22.29	13	22	2
Diptera	*Drosophila melanogaster*	19,517	17.84	13	22	2
Diptera	*Drosophila mojavensis*	14,904	23.54	13	22	2
Diptera	*Drosophila simulans *KY007	14,946	22.33	13	22	2
Diptera	*Drosophila simulans *KY045	14,946	22.36	13	22	2
Diptera	*Drosophila simulans *KY201	14,946	22.32	13	22	2
Diptera	*Drosophila simulans *KY215	14,946	22.33	13	22	2
Diptera	*Drosophila persimilis*	14,930	22.70	13	22	2
Diptera	*Drosophila virilis*	14,949	23.25	13	22	2
Diptera	*Drosophila willistoni*	14,915	22.76	13	22	2
Diptera	*Drosophila yakuba*	16,019	21.41	13	22	2
Diptera	*Simosyrphus grandicornis*	16,141	19.16	13	22	2
Diptera	Stomoxys calcitrans*	16,790	21.07	12	23	2
Diptera	*Trichophthalma punctata*	16,396	26.04	13	21	2

Hymenoptera	*Abispa ephippium*	16,953	19.39	13	26	2
Hymenoptera	*Apis mellifera*	16,343	15.14	13	22	2
Hymenoptera	*Bombus ignites*	16,434	13.22	13	22	2
Hymenoptera	Vanhornia eucnemidarum*	16,574	19.86	13	18	2
Hymenoptera	Xenos vesparum*	14,519	20.68	13	23	1

Lepidoptera	*Adoxophyes honmai*	15,680	19.61	13	22	2
Lepidoptera	*Bombyx mandarina*	15,928	18.32	13	22	2
Lepidoptera	*Bombyx mori *C-108	15,656	18.64	13	22	2
Lepidoptera	*Coreana raphaelis*	15,314	17.34	13	23	2
Lepidoptera	*Manduca sexta*	15,516	18.21	13	23	2
Lepidoptera	*Ochrogaster lunifer*	15,593	22.16	13	22	2
Lepidoptera	Ostrinia furnacalis*	14,536	19.62	13	22	2
Lepidoptera	Ostrinia nubilalis*	14,535	19.84	13	22	2
Lepidoptera	*Saturnia boisduvalii*	15,360	19.38	13	22	2

Megaloptera	*Corydalus cornutus*	15,687	25.10	13	22	2
Megaloptera	*Protohermes concolorus*	15,851	24.17	13	22	2

Neuroptera	*Ascaloptynx appendiculatus*	15,877	24.43	13	22	2
Neuroptera	*Polystoechotes punctatus*	16,036	21.04	12	22	2

*Nearly completely sequenced mitochondrial genome.

**Table 3:** List of 76 whole mitochondrial genomes of hexapod species (Part II. 73 species excluding holometabolous orders) archived in IMGD

**Order**	**Species**	**Size (bp)**	**GC (%)**	**PCGs**	**tRNAs**	**rRNAs**
Archaeognatha	*Nesomachilis australica*	15,474	31.17	13	21	2
Archaeognatha	*Pedetontus silvestrii*	15,879	25.66	13	22	2
Archaeognatha	*Petrobius brevistylis*	15,698	32.12	13	22	2
Archaeognatha	*Trigoniophthalmus alternatus*	16,197	28.59	13	22	2

Zygentoma	*Thermobia domestica*	15,152	33.01	13	22	2
Zygentoma	*Tricholepidion gertschi*	15,267	31.40	13	22	2

Collembola	*Cryptopygus antarcticus*	15,297	29.10	13	23	2
Collembola	*Gomphiocephalus hodgsoni*	15,075	25.92	13	22	2
Collembola	*Friesea grisea*	15,425	27.73	13	22	2
Collembola	Onychiurus orientalis*	12,984	30.89	13	20	1
Collembola	*Orchesella villosa*	14,924	27.82	13	22	2
Collembola	Podura aquatica*	13,809	34.21	13	20	1
Collembola	*Sminthurus viridis*	14,817	30.56	13	22	2
Collembola	*Tetrodontophora bielanensis*	15,455	27.32	13	22	2

Diplura	*Campodea fragilis*	14,965	27.44	13	22	2
Diplura	*Campodea lubbocki*	14,974	25.19	13	22	2
Diplura	*Japyx solifugus*	15,785	35.18	13	22	2

Ephemeroptera	*Parafronurus youi*	15,481	33.62	13	23	2

Odonata	Orthetrum triangulare melania*	14,033	26.09	13	19	2

Grylloblattodea	Grylloblatta sculleni*	15,595	29.71	12	19	2

Blattaria	*Periplaneta fuliginosa*	14,996	24.85	13	22	2

Isoptera	*Reticulitermes flavipes *IS13	16,565	33.82	13	22	2
Isoptera	*Reticulitermes flavipes *IS57	16,569	33.76	13	22	2
Isoptera	*Reticulitermes flavipes *IS58	16,567	33.78	13	22	2
Isoptera	*Reticulitermes hageni*	16,590	34.45	13	22	2
Isoptera	*Reticulitermes santonensis *IS54	16,567	33.91	13	22	2
Isoptera	*Reticulitermes virginicus *IS59	16,513	34.12	13	22	2
Isoptera	*Reticulitermes virginicus *IS60	15,966	34.37	13	22	2

Mantodea	*Tamolanica tamolana*	16,055	24.73	13	22	2

Mantophasmatodea	*Sclerophasma paresisense*	15,500	24.94	13	22	2

Orthoptera	*Acrida willemsei*	15,601	23.78	13	22	2
Orthoptera	*Anabrus simplex*	15,766	30.56	13	22	2
Orthoptera	*Calliptamus italicus*	15,675	26.74	13	22	2
Orthoptera	*Chorthippus chinensis*	15,599	24.89	13	22	2
Orthoptera	*Deracantha onos*	15,650	30.76	13	22	2
Orthoptera	*Gryllotalpa orientalis*	15,521	29.51	13	22	2
Orthoptera	*Gryllotalpa pluvialis*	15,525	27.80	13	22	2
Orthoptera	*Locusta migratoria*	15,722	24.67	13	22	2
Orthoptera	*Myrmecophilus manni*	15,323	29.82	13	22	2
Orthoptera	*Oxya chinensis*	15,443	24.11	13	22	2
Orthoptera	*Ruspolia dubia*	14,971	29.14	13	22	2
Orthoptera	*Gastrimargus marmoratus*	15,924	24.82	13	22	2
Orthoptera	*Gampsocleis gratiosa*	15,929	34.69	13	22	2
Orthoptera	*Troglophilus neglectus*	15,810	26.63	13	23	2

Phasmatodea	Timema californicum*	14,387	27.86	13	19	1

Plecoptera	*Pteronarcys princeps*	16,004	28.54	13	22	2

Hemiptera	Aeschyntelus notatus*	14,532	24.29	13	22	2
Hemiptera	*Aleurochiton aceris*	15,388	22.10	13	21	2
Hemiptera	*Aleurodicus dugesii*	15,723	13.67	13	20	2
Hemiptera	*Bemisia tabaci*	15,322	24.32	13	22	2
Hemiptera	*Coptosoma bifaria*	16,179	28.67	13	22	2
Hemiptera	*Dysdercus cingulatus*	16,249	22.31	13	22	2
Hemiptera	Geocoris pallidipennis*	14,592	24.14	13	22	2
Hemiptera	*Hydaropsis longirostris*	16,521	24.54	13	22	2
Hemiptera	Macroscytus subaeneus*	14,620	26.21	13	22	2
Hemiptera	*Malcus inconspicuus*	15,575	22.20	13	22	2
Hemiptera	*Neomaskellia andropogonis*	14,496	18.73	13	18	2
Hemiptera	*Neuroctenus parus*	15,354	31.14	13	22	2
Hemiptera	*Nezara viridula*	16,889	23.12	13	22	2
Hemiptera	Orius niger*	14,494	23.47	13	22	2
Hemiptera	*Pachypsylla venusta*	14,711	25.00	13	22	2
Hemiptera	Phaenacantha marcida*	14,540	26.54	13	22	2
Hemiptera	*Philaenus spumarius*	16,324	23.01	13	22	2
Hemiptera	*Physopelta gutta*	14,935	25.49	13	22	2
Hemiptera	*Riptortus pedestris*	17,191	23.41	13	22	2
Hemiptera	*Saldula arsenjevi*	15,324	25.39	13	22	2
Hemiptera	*Schizaphis graminum*	15,721	16.06	13	22	2
Hemiptera	*Tetraleurodes acacia*	15,080	28.02	13	19	2
Hemiptera	*Trialeurodes vaporariorum*	18,414	27.70	13	22	2
Hemiptera	*Triatoma dimidiate*	17,019	30.43	13	22	2
Hemiptera	*Yemmalysus parallelus*	15,747	22.82	13	22	2

Phthiraptera	*Bothriometopus macrocnemis*	15,564	29.20	13	25	2
Phthiraptera	*Campanulotes bidentatus*	14,804	29.88	13	22	2
Phthiraptera	*Heterodoxus macropus*	14,670	20.72	13	22	2

Psocoptera	Lepidopsocid sp. RS2001	16,924	20.98	13	22	2

Thysanoptera	*Thrips imaginis*	15,407	23.43	13	23	2

*Nearly completely sequenced mitochondrial genome.

## Construction and content

### System architecture and design

**Figure 1:**
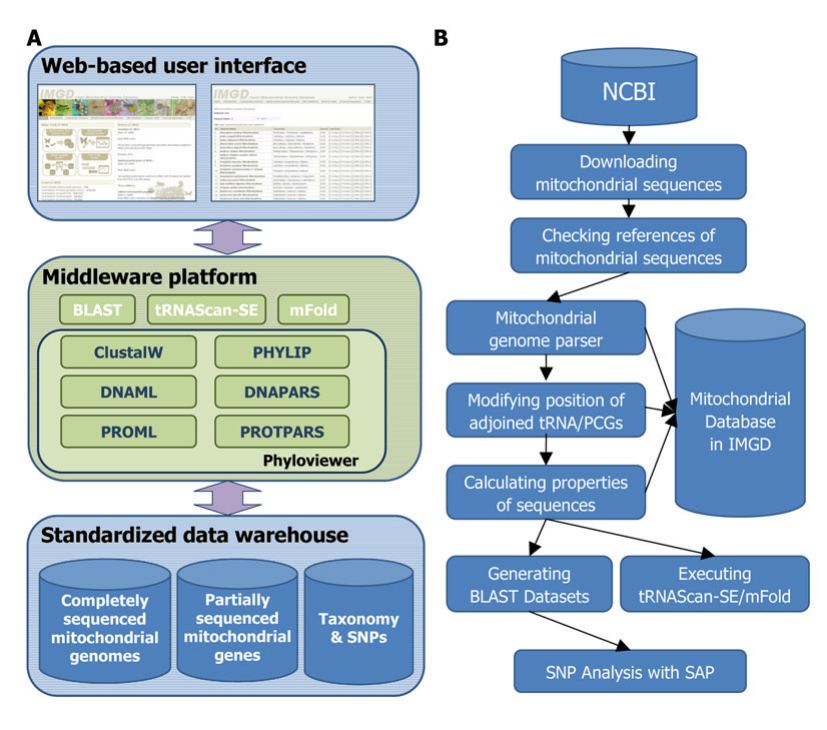
**The system architecture and pipeline of IMGD**. (A) Each rectangular box shows three layers. In the standardized data warehouse, diverse databases are placed. The middleware platform manages not only BLAST, tRNAScan-SE, and mFold but also six phylogenetic tools managed by Phyloviewer (). The web-based user interface supports browsing all information deposited in IMGD. (B) The pipeline for archiving hexapod mitochondrial sequences and calculating their properties was presented as a flowchart diagram.

The IMGD consists of three integrated layers: i) a standardized data warehouse that is supported by CFGP [24], ii) the middleware platform for the integration of various bioinformatics programs via standardized input and output interfaces, and iii) the web-based user interface, including the Species-driven User Interface (Figure 1A). In order to support the efficient archiving and analysis of a very large number of heterogeneous mitochondrial gene sequences (Table 2 and Table 3), a standardized structure for sequence data was required: this requirement was solved using CFGP [24], which has demonstrated its reliability and expandability via several published databases [32-37].

To support phylogenetic studies using the archived hexapod mitochondrial sequences, ClustalW (Version 1.83), PHYLIP (Version 3.68), and PHYML (Version 3.0) [29-31], which support the Neighbour Joining (NJ), Maximum Parsimony (MP), and Maximum Likelihood (ML) methods, respectively, were incorporated. The visualization and management of the resulting phylogenetic data are supported by the Phyloviewer (), which has been successfully employed in other platforms [24,34,35]. BLAST [26] was integrated with datasets containing mitochondrial gene sequences and hexapod taxonomy information, and tRNAscan-SE (Version 1.23) [27] and mFold (Version 3.2) [28] were embedded to allow for the display and comparison of secondary structures of tRNAs and anticodon sequences.

The user interface of IMGD provides the Mitochondrial Genome Browser, which is founded on the SNU Genome Browser () [25], to support the browsing and comparison of mitochondrial genome sequences in both the text and graphical forms via an interactive interface, and the Partial Sequence Browser to allow for the browsing of partially sequenced mitochondrial sequences. The IMGD also provides the Object Browser, which can collect and move selected sequences in IMGD into the Favorite, a personalized virtual storage space, for further data analyses using the analysis tools in both IMGD and CFGP [24]. The IMGD archives sequences and taxonomical information from more than 25,000 hexapod species. To facilitate the organization and presentation of data according to the taxonomic position/grouping of the species of origin, a new interface named the Species-driven User Interface (SUI) was designed and implemented in IMGD.

### Pipeline for updating the IMGD data warehouse

To support periodic updating of the IMGD data warehouse, the following automatic analysis pipeline was developed (Figure 1B). In the first step, completely and partially sequenced mitochondrial genome sequences are downloaded from NCBI using proper keywords after filtering out unpublished sequences. The downloaded sequences are subsequently filtered using several stop words in order to remove non-mitochondrial sequences. Secondly, the mitochondrial genome parsers, which were written in PERL, parse and store the filtered data into the data warehouse. Thirdly, adjoined stop codons at the 3'-end of the PCGs that overlap with neighboring PCGs or tRNAs in the mitochondrial genome [38], are manually checked to determine whether they are correct or not. Lastly, certain properties of the genome, including the CG content, AT skew, and codon usage, are calculated for graphical representations via SNU Genome Browser, and various cache tables are updated. In the final step, BLAST datasets, tRNA annotation information via both tRNAScan-SE [27] and mFold [28], and SNP databases are updated.

### Taxonomic origins of the sequences data archived in IMGD

The IMGD archives 132 hexapod mitochondrial genomes and 113,985 GenBank accessions of partially sequenced mitochondrial genes, consisting of 102,430 PCGs, 19,452 rRNAs, and 17,944 tRNAs, from 25,747 species belonging to 33 orders (Table 1). More than 10,000 mitochondrial gene sequences were derived from >1,000 species in the orders Coleoptera, Lepidoptera, Hymenoptera, and Diptera. In particular, members of Diptera and Coleoptera account for 26 (20.00%) and 12 (9.23%) mitochondrial genomes, respectively, reflecting active researches on these orders [39,40]. In contrast, the following 13 orders (39.39%) are represented only by less than 50 species in total: Dermaptera, Siphonaptera, Zygentoma, Grylloblattodea, Mantophasmatodea, Embioptera, Diplura, Archaeognatha, Strepsiptera, Megaloptera, Raphidioptera, Protura, and Zoraptera (Table 1). The underrepresentation of mitochondrial gene sequences from many orders suggests that to adequately support the analysis of evolutionary relationships within the Hexapoda, these underrepresented orders require more attention.

### Notable features in hexapod mitochondrial genomes

**Figure 2:**
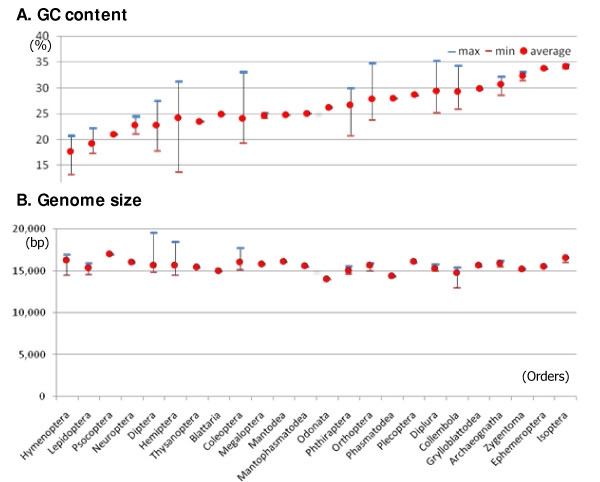
**Estimates of the GC content and genome size of the 132 hexapod mitochondrial genomes**. (A) The ranges of the GC content in the nearly completely and the completely sequenced mitochondrial genomes are shown. The closed red circle indicates the average GC content, and the blue and red bars present the maximum and minimum GC contents, respectively. (B) The distribution of mitochondrial genome sizes in different hexapod orders is shown. The closed red circle indicates the average mitochondrial genome size and the blue and red bars present the largest and smallest genome sizes, respectively (*see also Table 2 and Table 3*).

The genome size, GC content, and the number of PCGs, tRNAs, and rRNAs of the 132 mitochondrial genomes archived in IMGD (Table 2 and Table 3) were assessed (Figure 2). The GC content ranges from 13.22% to 35.18% with an average of 25.09%, showing the association at the order level (Figure 2A). The genome sizes vary from 12,984 bp to 19,517 bp, with an average of 15,617 bp with no clear correlation at any taxon levels (Figure 2B). Analyses of gene order in the 112 completely sequenced mitochondrial genomes revealed several interesting features. In 42 genomes (37.50%), which represent 12 orders, at least 222 gene insertions, deletions, inversions, and translocations were identified relative to the gene arrangement of the ancestral insect *Drosophila yakuba *[3,41] (Lee *et al*., in preparation). Gene translocations and inversions were detected in the following 12 orders: Collembola, Archaeognatha, Zygentoma, Hemiptera, Thysanoptera, Psocoptera, Phthiraptera, Neuroptera, Hymenoptera, Orthoptera, Lepidoptera, and Diptera. Gene insertions and deletions were detected in Collembola, Ephemeroptera, Orthoptera, Hemiptera, Phthiraptera, Diptera, and Lepidoptera.

### Examples of phylogenetic analyses results using insectmitochondrial genomes

**Figure 3:**
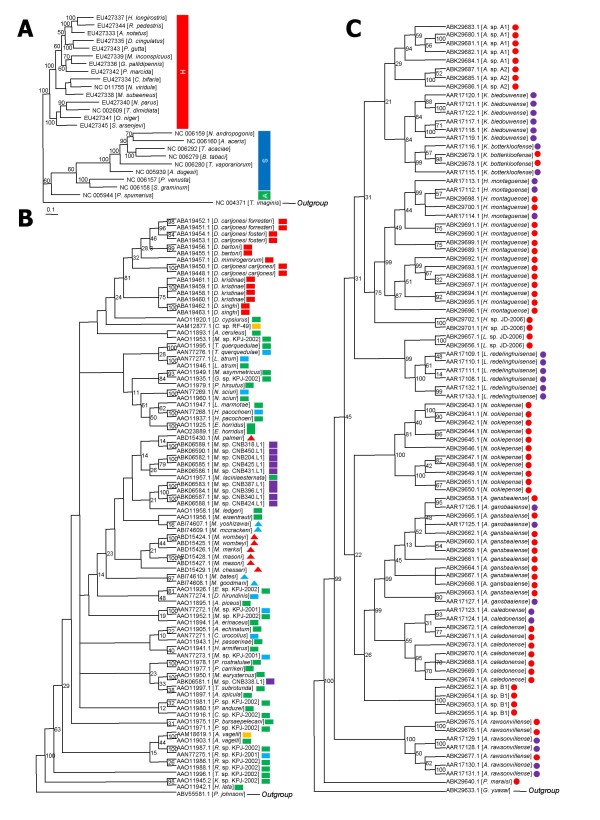
**Examples of phylogenetic analyses conducted using data and tools in IMGD**. (A) ML tree of the 24 Hemipteran species (19 completely and 5 nearly completed mitochondrial genomes) with *Thrips imaginis *(Thysanoptera) as an outgroup was constructed using DNAML. S, Sternorrhyncha; A, Auchenorrhyncha; H, Heteroptera. (B) MP tree built based on 88 *COI *sequences from 70 Phthirapteran species using DNAPARS, is shown. *Ptycta johnsoni *(Psocoptera) was used as an outgroup. The blue square indicates the sequences originated from Johnson and Whiting (2002) [42]; green square, Johnson *et al*. (2003) [43]; blue triangle, Price and Johnson (2006) [44]; red, violet and yellow squares, and red triangle present unpublished mitochondrial gene sequences. (C) MP tree using 90 *COI *sequences from 14 Mantophasmatodean species, with *Galloisiana yuasai *(Grylloblattodea) as an outgroup, was drawn using DNAPARS. The red circle indicates the mitochondrial sequences reported by Damgaard *et al*. (2008) [46] and violet circle presents the sequences from the study of Klass *et al*. (2003) [45]. The numbers on individual nodes of the trees in A, B, and C indicate bootstrap values with 10, 100, and 100 repeats, respectively, and the names of the species used and NCBI accession numbers are shown at the end of individual branches.

To demonstrate the utility of IMGD for phylogenetic analysis and also to test the system, many phylogenetic analyses using the data archived in IMGD have been conducted (e.g., Figure 3). Figure 3A shows an ML phylogenetic tree based on 19 completely sequenced and 5 nearly completed mitochondrial genomes in the order Hemiptera, which clearly shows two major suborder clades (Sternorrhyncha + Auchenorrhyncha and Heteroptera). The MP trees based on the *COI *gene sequences (Figure 3B and 3C) revealed more comprehensive phylogenetic relationships than those derived from previous studies in the orders Phthiraptera [42-44] and Mantophasmatodea [45,46].

### Single Nucleotide Polymorphisms among 9 insect mitochondrial genomes

**Figure 4:**
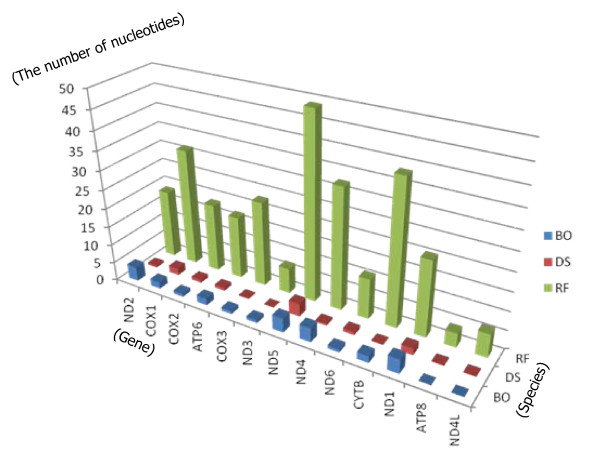
**Distribution of SNPs in 13 PCGs in 9 mitochondrial genomes**. The bar graph displays the distribution of SNPs in 13 PCGs of three insect species: BO, *Bactrocera oleae*; DS, *Drosophila simulans*; RF, *Reticulitermes flavipes. ATP6 *and *8 *(ATP synthase subunit 6 and 8); *COX1–3 *(cytochrome c oxidase subunits I–III); *CYTB *(cytochrome b); *ND1–6 *(NADH dehydrogenase subunits 1–6); *ND4L *(NADH dehydrogenase subunit 4L) (*see also Table 4*).

Single nucleotide polymorphisms (SNPs) in eight species with more than one mitochondrial genome having been sequenced (Table 4), were analyzed via the SNP Analysis Platform (), which is based on BLAST. A total of 856 SNPs and 30 insertion and deletions (INDELs) were identified (Table 4) from 187 kbp of aligned mitochondrial genome sequences (6 pair-wise comparisons of mitochondrial genomes). Among these, 621 SNPs (72.55%) were identified in 13 PCGs and designated as cSNPs. Figure 4 shows the average number of cSNPs in each species, order and PCG. *Bactrocera oleae *(BO), *Drosophila simulans *(DS), and *Reticulitermes flavipes *(RF) exhibited the highest frequency of cSNPs, similar to the results from previous genome sequence analyses [47-49]. Among the 13 PCGs, the *COI*, NADH dehydrogenase subunit 4 (*ND4*), and/or NADH dehydrogenase subunit 5 (*ND5*) genes showed the highest frequency of SNPs in Diptera (*COI *and *ND5*) and Isoptera (cytochrome b, *ND4*, and *ND5*) (Figure 4). These regions can serve as potential molecular markers in population genetic studies of these three orders.

**Table 4:** List of mitochondrial genome comparisons for SNP analysis

**Order**	**Source/Target Species**	**Size (bp)**	**Aligned (bp)**	**SNPs**	**INDELs**
Diptera	*Bactrocera oleae *Italy vs	15,815	15,815	31	0
	*Bactrocera oleae *portugal	15,815	15,815		
	
	*Drosophila simulans *KY007	14,946	14,946	25	2
	*Drosophila simulans *KY045	14,946	14,946		
	
	*Drosophila simulans *KY007	14,946	14,946	17	2
	*Drosophila simulans *KY201	14,946	14,946		
	
	*Drosophila simulans *KY007	14,946	14,946	6	0
	*Drosophila simulans *KY215	14,946	14,946		

Isoptera	*Reticulitermes flavipes *IS13 vs	16,565	16,561	393	14
	*Reticulitermes flavipes *IS57	16,569	16,565		
	
	*Reticulitermes flavipes *IS13 vs	16,565	16,561	384	12
	*Reticulitermes flavipes *IS58	16,567	16,563		

**Total**	6 pair-wise comparisons	187,572	187,556	856	30

## Utilities and discussion

### Species-driven User Interface (SUI)

**Figure 5:**
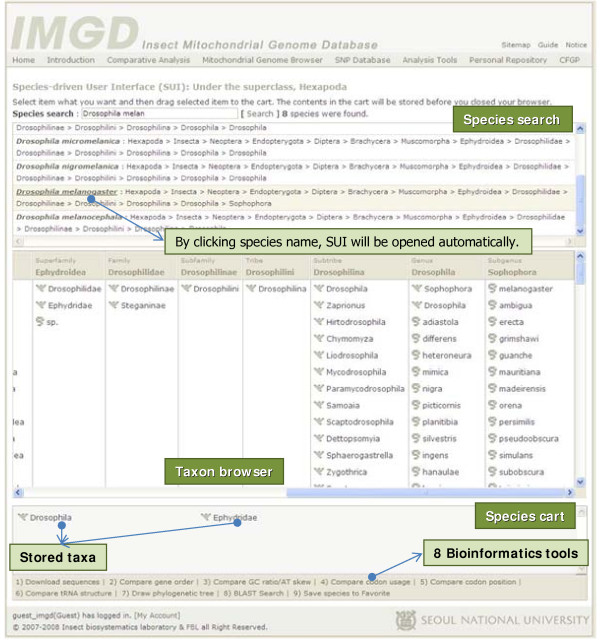
**Species-driven User Interface (SUI) optimized for collecting data based on taxa**. The Species-driven User Interface (SUI) consists of three parts: i) Species search, ii) Taxon browser, and iii) Species cart. The Species search function supports the search of species by species name. The Taxon browser provides the interface for browsing taxa in a hierarchical manner. The Species cart can store selected taxa, bridging the data from them to nine bioinformatics tools.

The SUI of IMGD supports efficient data retrieval and analysis at multi-taxon levels. The SUI was developed using Ajax technology, which supports faster performance than other methods (e.g. JavaScript and Java applets). The SUI helps the users of IMGD search hexapod taxa using the 'Species search' and supports the addition and deletion of selected insect species via the 'Species cart' function, which is similar to the cart functions commonly used on online shopping sites (Figure 3). After placing the taxa of interest in the cart, they can be analyzed in the following ways: i) downloading nucleotide and protein sequences and/or storing them into the Favorite with various options, ii) comparing gene orders, GC content/AT skew, codon usage and position among mitochondrial genomes, iii) displaying tRNA secondary structures predicted by tRNAScan-SE [27] and mFold [28], iv) executing ClustalW for multiple sequence alignment and calculating phylogenetic trees based on three methods, including NJ, MP, and ML, with a bootstrapping option, v) executing a BLAST search against the selected taxa, and vi) saving species information into the Favorite for future analyses (Figure 5). Since SUI was designed using a standardized application programming interface (API), additional programs can be easily incorporated into SUI.

### Gene order browser for graphical presentation of elements on the mitochondrial genome

**Figure 6:**
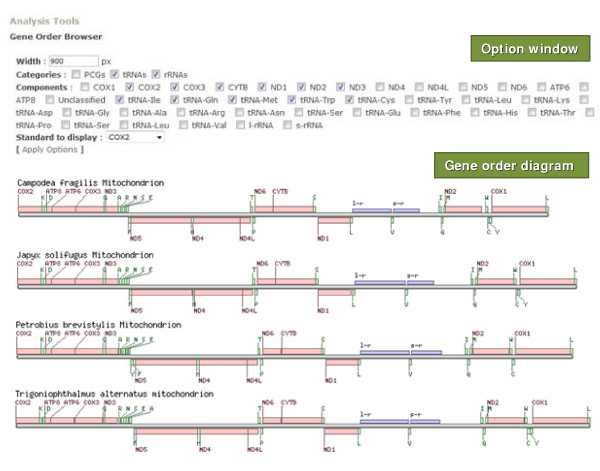
**Gene order browser for graphical presentation of the mitochondrial gene order**. The gene order browser consists of two parts: one is the option window and the other is the gene order diagram. In the option window, three options, including width, categories, and components, are displayed. After clicking 'Apply Options,' a gene order diagram based on the chosen option will be displayed. To indicate the nature of specific genetic elements on displayed mitochondrial genomes, the following abbreviations were used: A, tRNA-Ala;C, tRNA-Cys; D, tRNA-Asp; E, tRNA-Glu; F, tRNA-Phe; G, tRNA-Gly; H, tRNA-His; I, tRNA-Ile; K, tRNA-Lys; L, tRNA-Leu; M, tRNA-Met; N, tRNA-Asn; P, tRNA-Pro; Q, tRNA-Gln; R, tRNA-Arg; S, tRNA-Ser; T, tRNA-Thr; V, tRNA-Val; W, tRNA-Trp; Y, tRNA-Tyr; COX1–3, cytochrome c oxidase subunits I–III; CYTB, cytochrome b; ATP6 and ATP8, subunits 6 and 8 of the F_0_ATPase; ND1–6 and nad4L, NADH dehydrogenase subunits 1–6 and 4L; l-r and s-r, large and small subunit of ribosomal RNA genes; PCGs, protein coding genes; rRNAs, ribosomal RNA genes; tRNAs, transfer RNA genes.

Gene rearrangement events in the mitochondrial genomes can be used for tracing the evolutionary history of the mitochondrial genomes in Hexapoda (Lee *et al*., in preparation). The gene order browser implemented in IMGD was designed for efficient graphical presentation of PCGs, tRNAs, and rRNAs in the mitochondrial genome. To display different features on the genome graphically, the browser uses three different colors for PCGs, tRNAs, and rRNAs, and presents names of individual units (Figure 6). Moreover, the gene order browser displays the gene organizations using a specific gene as the start site for the linear genome diagrams regardless of the arbitrary start position given to individual mitochondrial genomes. Users can choose the number of mitochondrial genomes to be displayed by selecting them via SUI.

### Integrated platform for phylogenetic analyses supported by Phyloviewer

The Phyloviewer () provides four phylogenetic analysis programs (ClustalW, DNAPARS/PROTPARS, DNAML/PROML, and PHYML [29-31]) via a common interface to support phylogenetic studies based on the mitochondrial gene/genome sequences archived in IMGD. Three different methods of drawing phylogenetic trees (NJ, MP, and ML) are currently available. In addition, the interactive capability of graphical presentation of sequence alignments and selecting and storing all sequences under a selected node in the resulting phylogenetic tree by clicking the node is also provided.

### Comparative mitochondrial genomics via the SNU Genome Browser

**Figure 7:**
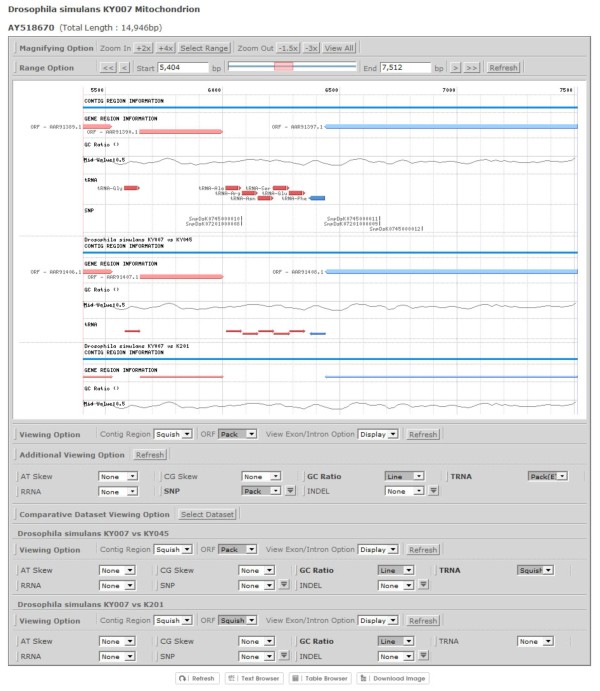
**Interactive graphical interface for visualizing aligned mitochondrial genomes via the SNU Genome Browser**. The SNU Genome Browser displays SNPs/INDELs as well as PCGs, tRNAs, GC contents among the aligned genomes of *Drosophila simulans *KY007, KY045, and K201 strains.

To support intuitive visualization of sequences, SNPs, and INDELs between two mitochondrial genomes, the SNU Genome Browser ()[25] was implemented. This recently developed genome browser provides an interactive user interface that supports visualization of the alignment region between genomes with the capability of comparing multiple genomes simultaneously (Figure 7). It also supports the text browser function for displaying nucleotide sequences of a selected region, supporting the confirmation of SNPs and INDELs. The table browser provides a list of individual elements present in the selected region with their positional information in a tabular form.

### Favorite, a personalized virtual space for storing data and conducting further analysis

**Figure 8:**
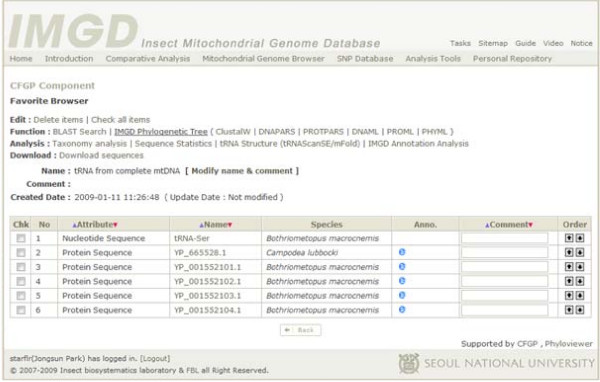
**Favorite, a personalized virtual space for data storage and further analyses**. The browser in Favorite provides four options: 'Edit,' 'Function,' 'Analysis,' and 'Download.' Any sequences listed at the bottom part can be selected by users for analyzing the selected sequences using seven programs and four analysis tools via the web.

Most of the data analysis and/or retrieval interfaces in IMGD provide the Object Browser, which allows users to save all or selected sequences and/or species shown into Favorite. This will help users manage their own datasets via IMGD. Through the interface of Favorite, BLAST, six different phylogenetics programs, and four data analysis tools are available for further analyses (Figure 8). The Favorite is linked to CFGP (), which provides not only diverse bioinformatic tools but also a data warehouse containing complete sequences of 19 insect nuclear genomes [24], so that further analyses with diverse resources can be conducted easily via this web interface.

## Conclusion

We developed IMGD to support versatile comparative analyses of hexapod mitochondrial gene/genome sequences. In IMGD, 132 completely or nearly-completely sequenced mitochondrial genomes and 113,985 mitochondrial gene sequences from 25,747 species were archived. The IMGD provides a variety of phylogenetic analysis tools via Phyloviewer, which supports the interactive graphical presentation of resultant phylogenetic trees. The IMGD, supported by the SNP analysis platform and the SNU Genome Browser, provides a graphical view of mitochondrial genome comparisons. In the near future, additional analysis tools, such as PAML [50] for the determination of positive/negative selection based on dS/dN values, will be integrated into IMGD. Moreover, based on the database of widely sequenced mitochondrial genes, an insect species identification system based on multiple loci can be developed. The IMGD is expected to significantly enhance evolutionary studies on the superclass Hexapoda using rapidly accumulating insect mitochondrial genome sequences.

## Availability and requirements

All data described in this paper can be browsed and downloaded through the IMGD web site at .

## Authors' contributions

WL, JP, SL, and YHL designed and managed this project, JC, JP, and WL construct the IMGD sequence database and WL, JL, JC, and JP curated all sequences in IMGD. KJ designed the IMGD web site and developed SUI. BP developed the interface for Phyloviewer, JP integrated SAP to IMGD and DH conducted mitochondrial genome alignments. JP, JC, BP, KJ, KA, DH and WS constructed and tested the whole web pages. WL, JP and SK wrote the manuscript.
